# Molecular and functional characterization of a typical 2-Cys peroxiredoxin and its developmental and pyriproxyfen-responsive expression in *Aedes albopictus* (Diptera: Culicidae)

**DOI:** 10.1093/jisesa/ieag119

**Published:** 2026-10-27

**Authors:** Batuhan Askim Arslanhan, Samba Deguene Diop, Simge Sahin, Ahsen Meliha Toroslu, Alina Denis Kizgin, Gokmen Zafer Pekmezci, Abdullah Inci, Alparslan Yildirim

**Affiliations:** Department of Parasitology, Faculty of Veterinary Medicine, Erciyes University, Melikgazi, Kayseri, Türkiye; Department of Parasitology, Faculty of Veterinary Medicine, Erciyes University, Melikgazi, Kayseri, Türkiye; Department of Parasitology, Faculty of Veterinary Medicine, Erciyes University, Melikgazi, Kayseri, Türkiye; Department of Parasitology, Faculty of Veterinary Medicine, Ondokuz Mayıs University, Atakum, Samsun, Türkiye; Department of Parasitology, Faculty of Veterinary Medicine, Erciyes University, Melikgazi, Kayseri, Türkiye; Department of Preclinical Sciences, Faculty of Veterinary Medicine, Ondokuz Mayıs University, Atakum, Samsun, Türkiye; Department of Parasitology, Faculty of Veterinary Medicine, Erciyes University, Melikgazi, Kayseri, Türkiye; Department of Parasitology, Faculty of Veterinary Medicine, Erciyes University, Melikgazi, Kayseri, Türkiye

**Keywords:** oxidative stress, thioredoxin system, juvenile hormone signaling, xenobiotic response, mosquito physiology

## Abstract

The Asian tiger mosquito, *Aedes albopictus* (Skuse, 1894) (Diptera: Culicidae), is established and expanding across the Black Sea region, but the redox processes associated with its development and response to insect growth regulators remain poorly understood. We characterized a typical 2-Cys peroxiredoxin (*Prx2*) by examining intraspecific sequence variation, conserved protein features, enzymatic activity, developmental transcription, and transcription following exposure to 0.06 ppb pyriproxyfen. The coding region was polymorphic among Turkish isolates, but all substitutions were synonymous, and the deduced amino acid sequence was conserved. Catalytic cysteines and hallmark motifs supported classification as a typical 2-Cys peroxiredoxin. Recombinant His-tagged *Prx2* migrated at the expected molecular size and removed hydrogen peroxide in a concentration- and thiol-dependent manner. Basal transcript abundance varied across development, reaching its lowest level in pupae and higher levels in adults. Exposure to 0.06 ppb pyriproxyfen was associated with increased *Prx2* transcript abundance in L3 and L4 larvae, whereas no statistically detectable treatment-related difference was observed among the surviving pupae or adults at the sampled time points. Together, these findings identify *Prx2* as a conserved, functional thiol peroxidase and demonstrate a pyriproxyfen-associated larval transcriptional response under the conditions tested. Because this concentration was not independently validated as sublethal in the present colony and later-stage sampling necessarily comprised survivors of the original exposure, the data do not distinguish developmental attenuation of the response from survivor selection.

## Graphical abstract

**Graphical Abstract:**
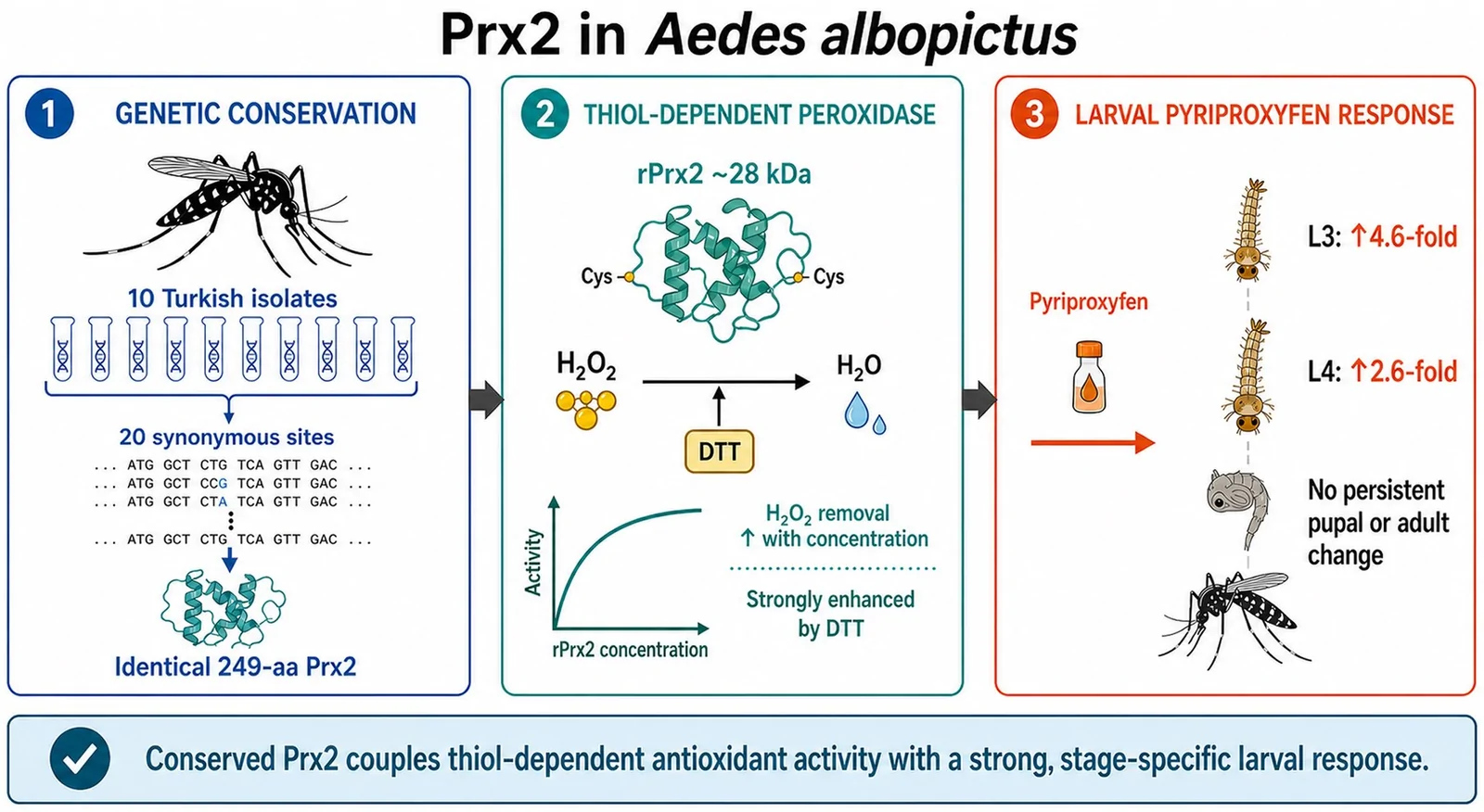

## Introduction

*Aedes albopictus* (Skuse, 1894) (Diptera: Culicidae) has expanded from its native Asian range into temperate and tropical regions worldwide and is an important vector of chikungunya, dengue, and Zika viruses (Benedict et al. 2007, Paupy et al. 2009, Bonizzoni et al. 2013). In Türkiye, the species was first recorded in the northwestern part of the country in 2011 and was subsequently documented across the Black Sea region (Oter et al. 2013, Akiner et al. 2016, Demirci et al. 2021). Recent field-based distribution modeling has identified additional suitable coastal and high-elevation habitats in the broader Black Sea region, while mitogenomic evidence indicates that Turkish populations contain multiple maternal lineages, consistent with more than one introduction event (Gunay et al. 2025, Diop et al. 2026). Its ecological plasticity, desiccation-resistant eggs, and ability to exploit abundant natural and artificial containers facilitate establishment but also complicate conventional control. Understanding the molecular mechanisms that support development and stress tolerance is therefore particularly relevant in recently established populations.

Reactive oxygen species (ROS) are generated during normal aerobic metabolism and may increase in response to immune activation, xenobiotic exposure, environmental stress, and extensive tissue remodeling. Although ROS participate in cellular signaling and host defense, their excessive accumulation can damage proteins, lipids, and nucleic acids. Peroxiredoxins (Prxs) are abundant cysteine-dependent peroxidases that regulate intracellular peroxide concentrations by reducing hydrogen peroxide, organic hydroperoxides, and related oxidants and can also participate in redox signaling (Wood et al. 2003, Hall et al. 2009, Perkins et al. 2015). In typical 2-Cys Prxs, the peroxidatic cysteine reacts with peroxide to form a sulfenic-acid intermediate, which subsequently forms an intermolecular disulfide bond with the resolving cysteine of the opposing subunit. The oxidized enzyme is then returned to its reduced, catalytically competent state by the thioredoxin system. Conserved structural elements, including the GGLG and C-terminal YF motifs, contribute to active-site organization and influence susceptibility to hyperoxidation (Hall et al. 2009; Perkins et al. 2015).

Studies in insects and other arthropods indicate that Prxs contribute not only to peroxide detoxification but also to development, reproduction, blood feeding, immune responses, and adaptation to environmental or chemical stress (Peterson and Luckhart 2006, Yu et al. 2011, Kusakisako et al. 2016, Yang et al. 2025). In *Anopheles stephensi*, a typical 2-Cys Prx protects mosquito cells against oxidative and nitrosative stresses associated with malaria parasite infection (Peterson and Luckhart 2006). In the hard tick *Haemaphysalis longicornis*, depletion of Prx2 increases hydrogen peroxide accumulation and impairs blood feeding and reproduction (Kusakisako et al. 2016). These findings demonstrate the physiological importance of arthropod Prxs, but the intraspecific coding variation, biochemical activity, and developmental expression of Prx2 remain insufficiently characterized in *Ae. albopictus*.

Pyriproxyfen is a juvenile hormone analog used to control immature mosquitoes, particularly through inhibition of pupal development and adult emergence, and has also been deployed in autodissemination strategies targeting cryptic *Ae. albopictus* larval habitats (Chandel et al. 2016). Its activity is mediated through juvenile-hormone signaling involving the Methoprene-tolerant receptor, and exposure can alter endocrine and metabolic gene-expression networks (Ahmed et al. 2020). Importantly, mosquitoes that survive nonlethal exposure may exhibit changes in reproductive performance, hormone-responsive transcription, or insecticide susceptibility (Opiyo et al. 2021, Kancharlapalli and Brelsfoard 2024). These findings raise the possibility that pyriproxyfen-induced endocrine and metabolic disruption also changes cellular redox requirements. However, this connection should be treated as a testable hypothesis because a direct pyriproxyfen-induced oxidative response involving Prx2 has not been demonstrated in *Ae. albopictus*.

In the present study, we characterized complete *Prx2* coding sequences from 10 Turkish *Ae. albopictus* isolates, examined conserved protein features and phylogenetic relationships, expressed and functionally evaluated recombinant *Prx2*, and quantified basal and pyriproxyfen-responsive transcription across developmental stages. We hypothesized that the catalytic architecture and amino acid sequence of *Prx2* would remain conserved despite intraspecific nucleotide variation and that exposure to 0.06 ppb pyriproxyfen would be associated with a developmentally variable transcriptional response.

## Materials and Methods

### Mosquito Material and Experimental Colony

Adult *Aedes albopictus* specimens representing 10 field isolates were obtained from nine provinces along the Black Sea and adjacent northwestern corridor of Türkiye: Bartın, Kastamonu, Artvin (two isolates), İstanbul, Kırklareli, Ordu, Sinop, Samsun, and Trabzon (Fig. 1). Specimens were identified morphologically using standard keys (Rueda 2004) and were retained for genomic DNA extraction. A laboratory colony derived from Trabzon was maintained to the F6 generation at 28 ± 1 °C, 70% ± 5% relative humidity, and a 12:12 (L:D) h photoperiod. Larvae were fed TetraMin fish food, and adults received 10% sucrose.

**Fig. 1.**
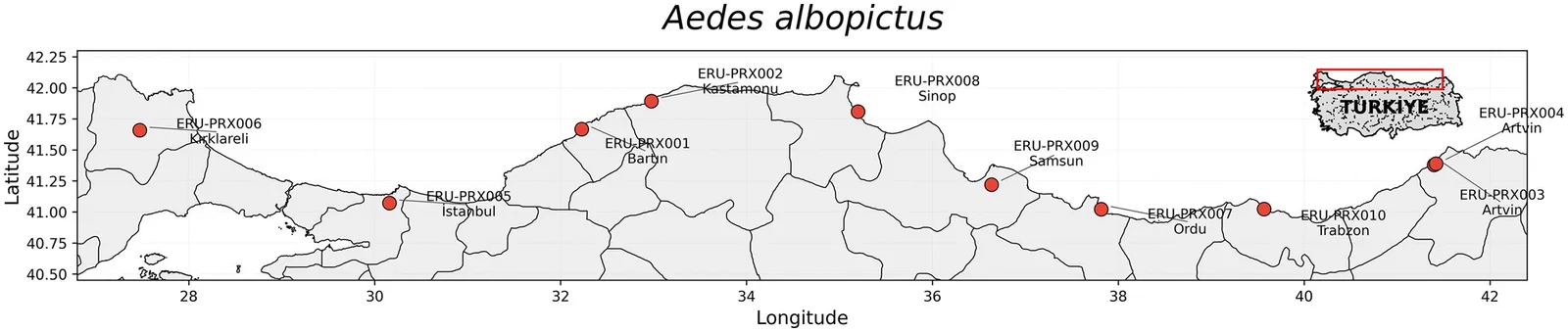
Sampling localities of *Aedes albopictus* isolates in Türkiye. Circles indicate the nine provinces represented by the 10 isolates used for molecular characterization: Bartın, Kastamonu, Artvin, İstanbul, Kırklareli, Ordu, Sinop, Samsun, and Trabzon.

### Shotgun Sequence Recovery and Quality Assessment

Genomic libraries were sequenced as paired-end reads on an Illumina NovaSeq 6000 platform. Raw reads were quality filtered with fastp v0.23.4 using 5′/3′ end trimming, a 4-bp sliding window with a mean-quality threshold of Q20, a minimum read length of 50 nt, and a maximum of five ambiguous bases per read; library-level read counts, base yields, mean read lengths, Q20/Q30 percentages, and GC content before and after filtering are reported in Supplementary Table S2A. Filtered reads were mapped with minimap2 v2.26-r1175 against the *Aedes albopictus* Prx2 reference transcript (XM_029870009.2) as the target, with the *Aedes albopictus* reference genome assembly (GCF_006496715.1) included to divert paralogous and off-target reads as decoys, and alignments were processed with SAMtools v1.19.2. Reads assigned to decoy sequences were excluded, duplicate reads were marked, and unique target reads with mapping quality (MAPQ) ≥ 30 were retained; read pairs anchored by these reads were recovered by pair-anchored rescue after decoy filtering. Base calling used duplicate-excluded reads with MAPQ ≥30 and base quality ≥30 at a minimum depth of 10×: a homozygous base was called when the major-allele frequency was ≥0.80, and a heterozygous position was called, and encoded with the appropriate IUPAC ambiguity code, when the minor allele was supported by at least three reads at a frequency ≥0.20, the two alleles together accounted for ≥0.90 of the reads, and both alleles had bidirectional read support. Positions that did not meet these criteria were not filled from the reference sequence. The 750-bp coding sequence was then extracted in the reference coordinate system, visually inspected, and translated to verify an uninterrupted open reading frame. Target- and decoy-mapping counts, duplicate rates, rescued reads, per-position CDS depth, and the number of strictly callable CDS positions for each isolate are reported in Supplementary Table S2B. The resulting sequences were deposited in GenBank under PZ829536–PZ829545, and isolate metadata are provided in Supplementary Table S1.

### Molecular Characterization and Phylogenetic Analysis

Coding sequences were aligned and translated using the standard genetic code. Variable sites, haplotypes, pairwise nucleotide identity, haplotype diversity, and nucleotide diversity were calculated across the complete 750-bp CDS alignment of the 10 isolates. Interspecific comparisons were restricted to the 516-nucleotide region encoding residues 57–228 (the typical 2-Cys peroxiredoxin homologous region) because the reference sequences differed markedly outside this region: the *Culex*, *Anopheles*, and *Toxorhynchites* RefSeq models encode predicted proteins of 615–662 amino acids in which the peroxiredoxin domain is preceded by long N-terminal extensions, whereas the aedine sequences have short N-terminal regions of differing length. This region could be aligned unambiguously, without insertions or deletions, across all 19 taxa. Protein properties of ERU-PRX001 were estimated with ExPASy ProtParam (Gasteiger et al. 2005). Similarity to the *Aedes albopictus* reference protein XP_029725869.1 and conserved domains were assessed using NCBI RefSeq and the Conserved Domain Database (O’Leary et al. 2016, Lu et al. 2020). The peroxidatic and resolving cysteines and the FTFVCPTEI, GGLG, EVCPAGW, and C-terminal YF motifs were inspected in the alignment.

Amino acid sequences spanning residues 57–228 of the typical 2-Cys peroxiredoxin homologous region were aligned with MAFFT v7 using the L-INS-i strategy (Katoh and Standley 2013). For nucleotide phylogeny, the corresponding 516-nucleotide codon alignment contained the 10 Turkish isolates and nine reference mosquito sequences. Maximum-likelihood inference was performed with IQ-TREE 2.0.7 (Minh et al. 2020). ModelFinder and partition merging selected TNe + I for codon positions 1 + 2 and TPM2 + F + G4 for position 3 (Chernomor et al. 2016, Kalyaanamoorthy et al. 2017). Branch support was estimated using 1,000 standard nonparametric bootstrap replicates (Felsenstein 1985) and summarized as an extended majority-rule consensus tree, with branch lengths optimized by maximum likelihood on the original alignment. Because the analysis was designed to verify the placement of the Turkish sequences rather than to resolve the root of mosquito Prx2 or the direction of sequence change, no taxon outside Culicidae was included, and an a priori outgroup was not imposed on this single-locus tree. The consensus tree was therefore midpoint-rooted for display only. Bootstrap values refer to bipartitions of the unrooted topology and are independent of root placement; after rerooting, each value was mapped to its corresponding bipartition rather than carried over as a node label (Czech et al. 2017). No inferences regarding ancestral states or evolutionary direction were based on the inferred root.

### Recombinant Prx2 Expression and Verification

The full-length *Prx2* coding sequence from ERU-PRX001 (GenBank accession no. PZ829536) was cloned into the pET-28a(+) expression vector and engineered to encode a C-terminal 6 × His tag. The resulting construct was introduced into *Escherichia coli* BL21(DE3) cells. Construct integrity was initially assessed by PCR using the T7 forward and gene-specific reverse primers and was subsequently confirmed by Sanger sequencing.

Bacterial cultures carrying the verified recombinant construct were induced with 0.1, 0.5, or 1.0 mM isopropyl β-D-1-thiogalactopyranoside (IPTG). Soluble recombinant protein was purified by nickel-affinity chromatography. Protein expression and enrichment were evaluated by Coomassie Brilliant Blue-stained SDS-PAGE, and the presence of the 6 × His-tagged recombinant product was confirmed by immunoblotting using an anti-6 × His antibody. The native ERU-PRX001 Prx2 polypeptide comprised 249 amino acids and had a predicted molecular mass of 28.115 kDa, excluding the C-terminal 6 × His tag and any vector-derived residues. Primer sequences are provided in Table 1.

**Table 1.** Primers used for recombinant construct preparation and verification and for quantitative real-time PCR analysis

Assay	Target/primer	Sequence (5′–3′)	Length (nt)	GC (%)	Tm (°C)	Product/purpose
**Construct preparation/verification**	Prx2F	TCAGAATTCATGCTGCCCCACCG	23	57	–	EcoRI-tailed forward primer
**Construct preparation/verification**	Prx2R	GTCAAGCTTCTAATATTTATGGTTCTTCTCGAAGTACT	38	34	–	HindIII-tailed reverse primer
**qPCR**	Prx2/2CYS-F	GGCAAGTATGTGGTGTTCTTCT	22	45.5	58.6	109-bp amplicon
**qPCR**	Prx2/2CYS-R	CGGGCGTTCAGCTTCCTAAAT	20	50.0	58.6	109-bp amplicon
**qPCR**	β-actin/ACT-F	TCTGACCGACTACCTGAT	18	50.0	53.7	130-bp amplicon
**qPCR**	β-actin/ACT-R	CATCTCCTGCTCGAAGTC	18	55.6	54.5	130-bp amplicon

Restriction sites in the construct primers are GAATTC (EcoRI) and AAGCTT (HindIII). GC, guanine–cytosine content; Tm, melting temperature; qPCR, quantitative real-time polymerase chain reaction.

### Hydrogen Peroxide-Removal Assay

Hydrogen peroxide-removal activity was measured with a ferrithiocyanate colorimetric assay adapted from Lim et al. (1993) and Yu et al. (2011). Reactions contained 100 mM HEPES (pH 7.0), 0, 5, 25, 50, 75, or 100 µg/ml recombinant Prx2, and either 10 mM dithiothreitol (DTT) or no DTT in a 100-µl volume. Mixtures were preincubated for 15 min at 37 °C, initiated with H2O2 at a final concentration of 20 µM, and incubated for an additional 15 min at 37 °C. Reactions were stopped with 75 µl of 12.5% (w/v) trichloroacetic acid. Residual peroxide was detected after addition of 50 µl 10 mM ammonium iron(II) sulfate and 100 µl 2.5 M potassium thiocyanate, and absorbance was read at 490 nm. Hydrogen peroxide removal was calculated as 100 × (A0 − Ax)/A0, where A0 is the no-protein control and Ax is the reaction containing Prx2. Three independent experiments were performed, each measured in technical triplicate.

### Developmental and Pyriproxyfen Exposure Experiments

Basal Prx2 transcription was evaluated across seven developmental stages of *Aedes albopictus*, including first-, second-, third-, and fourth-instar larvae (L1–L4), pupae, adult males, and adult females. Three independent biological replicates were collected for each developmental stage.

For the pyriproxyfen exposure experiment, larvae were maintained under the standard rearing conditions described above until completion of the second larval instar. At the transition from L2 to L3, newly molted L3 larvae were allocated to pyriproxyfen-treated and control groups. Each biological replicate was initiated with 60 larvae in both the pyriproxyfen and control groups, and three independent biological replicates were established per treatment. Thus, each treatment initially comprised 180 larvae distributed among three replicate containers.

Pyriproxyfen exposure was performed using an experimental approach adapted from Alto et al. (2025). The nominal concentration of 0.06 ppb was selected because it corresponded to an LC40 treatment in the previous *Ae. albopictus* bioassay, which was conducted under the same rearing conditions (28 °C, 14:10 h light:dark) used here (Alto et al. 2025). Susceptibility to pyriproxyfen was not independently characterized in the present F6 colony; therefore, the treatment is described by its nominal concentration rather than as a sublethal exposure. The commercial juvenile hormone analog formulation NyGuard IGR [2-(1-methyl-2-(4-phenoxyphenoxy)ethoxy)pyridine] was diluted directly in water to obtain a final pyriproxyfen concentration of 0.06 ppb in an assay volume of 400 ml per replicate container. No additional organic solvent was used. Control containers received an equivalent volume of water without pyriproxyfen. Experimental groups were maintained at 28 °C under a 14:10 h light:dark photoperiod throughout the experiment.

The first post-exposure sample was collected 24 h after pyriproxyfen application, while exposed individuals remained in the L3 stage. This interval was selected a priori to assess transcription after one day of exposure and was not intended to represent an experimentally determined peak response. No separate concentration-response or transcriptional time-course experiment was conducted. The remaining surviving individuals were allowed to continue development, and subsequent samples were collected 24 h after transition to the L4, pupal, adult-male, or adult-female stage. Consequently, later-stage samples represented individuals that survived and completed development after the initial treatment. These samples were used to determine whether treatment-associated transcriptional differences remained detectable at the specified sampling points; the design could not distinguish developmental attenuation from survivor selection.

### RNA Isolation, Reverse Transcription, and Quantitative PCR

Total RNA was isolated separately from each independent biological replicate using TRIzol Reagent (Invitrogen, Thermo Fisher Scientific, USA) according to the manufacturer’s instructions. For gene-expression analyses, individuals from the corresponding developmental stage were pooled to constitute each biological replicate. Specifically, 10 L1 larvae, eight L2 larvae, five L3 larvae, three L4 larvae, three pupae, and three adults were pooled per biological replicate for RNA extraction. Adult males and adult females were processed separately, with three individuals pooled per biological replicate for each sex. RNA concentration was measured using the Qubit RNA Assay, and purity was assessed spectrophotometrically. RNA integrity was not quantified using a formal integrity metric such as the RNA integrity number (RIN); agarose-gel or capillary-electrophoretic inspection was used when required. Three independent biological replicates were analyzed for every developmental stage and, in the pyriproxyfen experiment, for each treatment group.

Following TRIzol-based extraction, residual genomic DNA was removed by DNase treatment. First-strand cDNA was synthesized from the purified RNA using the ProtoScript II First Strand cDNA Synthesis Kit (New England Biolabs, USA) according to the manufacturer’s instructions. The resulting cDNA was used as a template for quantitative real-time PCR. Relative transcription of *Prx2* was quantified using *β-actin* as the sole reference gene for normalization. The stability of *β-actin* across developmental stages and pyriproxyfen treatment conditions was not independently evaluated in this experiment. Gene-specific primers targeting the *Prx2* coding sequence were designed from the sequence characterized in the present study. Primer sequences, GC contents, melting temperatures, and expected amplicon sizes are provided in Table 1.

qPCR reactions were performed using ClearPeak 2× SYBR Master Mix (EcoTech Biotechnology, Türkiye) on a CFX Connect Real-Time PCR Detection System (Bio-Rad Laboratories, Hercules, CA, USA). Each reaction was prepared in a final volume of 20 µl, containing 10 µl of ClearPeak 2× SYBR Master Mix, 0.8 µl each of forward and reverse primers (10 µM), 2 µl of cDNA template, and nuclease-free water to a final volume of 20 µl. Prx2 and β-actin were amplified in separate wells under identical thermal-cycling conditions.

Amplification was initiated at 95 °C for 30 s, followed by 40 cycles of 95 °C for 10 s and 60 °C for 30 s. Fluorescence was recorded during the annealing/extension step. Following amplification, melt-curve analysis was performed to assess amplification specificity, and reactions producing a single distinct melting peak were considered specific. No-template controls were included to monitor potential contamination and nonspecific amplification. A serial-dilution standard curve was not included in the analytical qPCR run; accordingly, amplification efficiencies and efficiency equivalence between the *Prx2* and *β-actin* assays could not be determined from the available instrument output. Reporting follows relevant MIQE items for which supporting measurements were available (Bustin et al. 2009).

For each biological replicate, *Prx2* expression was normalized to *β-actin* as ΔCq = Cq(*Prx2*) − Cq(*β-actin*). Relative transcript abundance for graphical presentation was calculated using the 2^−^ΔΔCq method (Livak and Schmittgen 2001). L1 was used as the calibrator for basal developmental expression, whereas pyriproxyfen-exposed samples were calibrated against the corresponding stage-matched water controls. Because the comparative Cq approach assumes comparable target and reference amplification efficiencies, which were not experimentally confirmed here, the reported fold-change estimates should be interpreted cautiously.

### Statistical Analysis

Statistical analyses were performed using SAS 9.4 (SAS Institute Inc. 2013). Independent biological or experimental replicates were treated as the observational units. Where technical measurements were performed, their mean was calculated before analysis, and technical replicates were not treated as independent observations. Unless otherwise indicated, results are presented as the mean ± SD of three independent replicates. Model assumptions were evaluated by examining residual normality and homogeneity of variance using the Shapiro–Wilk and Levene tests, respectively.

For gene-expression analyses, ΔCq values were calculated for each biological replicate as Cq(*Prx2*) − Cq(*β-actin*). Relative expression values were calculated using the 2^−^ΔΔCq method and were used for graphical presentation. To avoid the distributional distortion associated with exponentiation, all statistical analyses of gene-expression data were performed using the corresponding ΔCq values rather than the 2^−^ΔΔCq values (Rieu and Powers 2009).

Basal *Prx2* expression across developmental stages was analyzed by one-way analysis of variance (ANOVA), with developmental stage included as a fixed factor. When the overall ANOVA was significant, pairwise comparisons among developmental stages were performed using Tukey-adjusted tests.

The pyriproxyfen experiment was analyzed by two-way ANOVA, with developmental stage, treatment, and their interaction included as fixed effects. When the stage × treatment interaction was significant, the main effects were not interpreted independently. Instead, prespecified stage-specific comparisons between pyriproxyfen-exposed groups and their corresponding stage-matched controls were performed using contrasts derived from the two-way ANOVA model. The resulting *P values* for the five parallel stage-specific comparisons were adjusted using the Holm procedure (Holm 1979). Comparisons among developmental stages within a treatment group were performed using Tukey adjustment.

H_2_O_2_-removal activity was analyzed by two-way ANOVA, with protein concentration, DTT condition, and their interaction included as fixed effects. The protein-free control (0 µg/ml) was included as one of the six concentration levels. When the concentration × DTT interaction was significant, the effect of DTT was evaluated separately at each protein concentration, and concentration effects were evaluated within each DTT condition. Tukey adjustment was applied to comparisons among protein concentrations, whereas Holm adjustment was applied to parallel comparisons between the + DTT and − DTT conditions.

## Results

### Sequence Recovery and Intraspecific Variation

The complete *Prx2* coding sequence was recovered from all 10 *Aedes albopictus* isolates and deposited in GenBank under accession numbers PZ829536–PZ829545. Each coding sequence was 750 bp in length, contained an ATG start codon and a TAG stop codon, and encoded a 249-amino-acid protein. All 750 CDS positions met the strict consensus-calling criteria in every isolate; mean CDS depth ranged from 39.7× to 110.6×, and the minimum per-position depth was 10–15 × (Supplementary Table S2B). No ambiguous nucleotides, frameshifts, or premature stop codons were detected. Isolate metadata and GenBank accession numbers are provided in Supplementary Table S1, library-level sequencing and filtering statistics in Supplementary Table S2A, and Prx2-specific mapping and coverage metrics in Supplementary Table S2B.

Comparison of the complete 750-bp coding-sequence alignment identified 20 polymorphic nucleotide positions at CDS positions 39, 72, 105, 111, 129, 135, 144, 210, 312, 390, 393, 408, 426, 528, 573, 600, 666, 696, 708, and 747 (Fig. 2). All substitutions occurred at third codon positions and were synonymous. Each of the 10 isolates represented a distinct nucleotide haplotype, resulting in a haplotype diversity (*Hd*) of 1.000. Nucleotide diversity (π) was 0.01239. Because these values derive from a single coding locus and generally one individual per locality, they are presented as descriptive summaries of the sampled sequences rather than as estimates of population-level diversity. Pairwise CDS-to-CDS nucleotide identity among isolates ranged from 98.133% to 99.867%, with a mean of 98.761%. Despite this nucleotide-level variation, all isolates encoded an identical 249-amino-acid Prx2 protein.

**Fig. 2.**
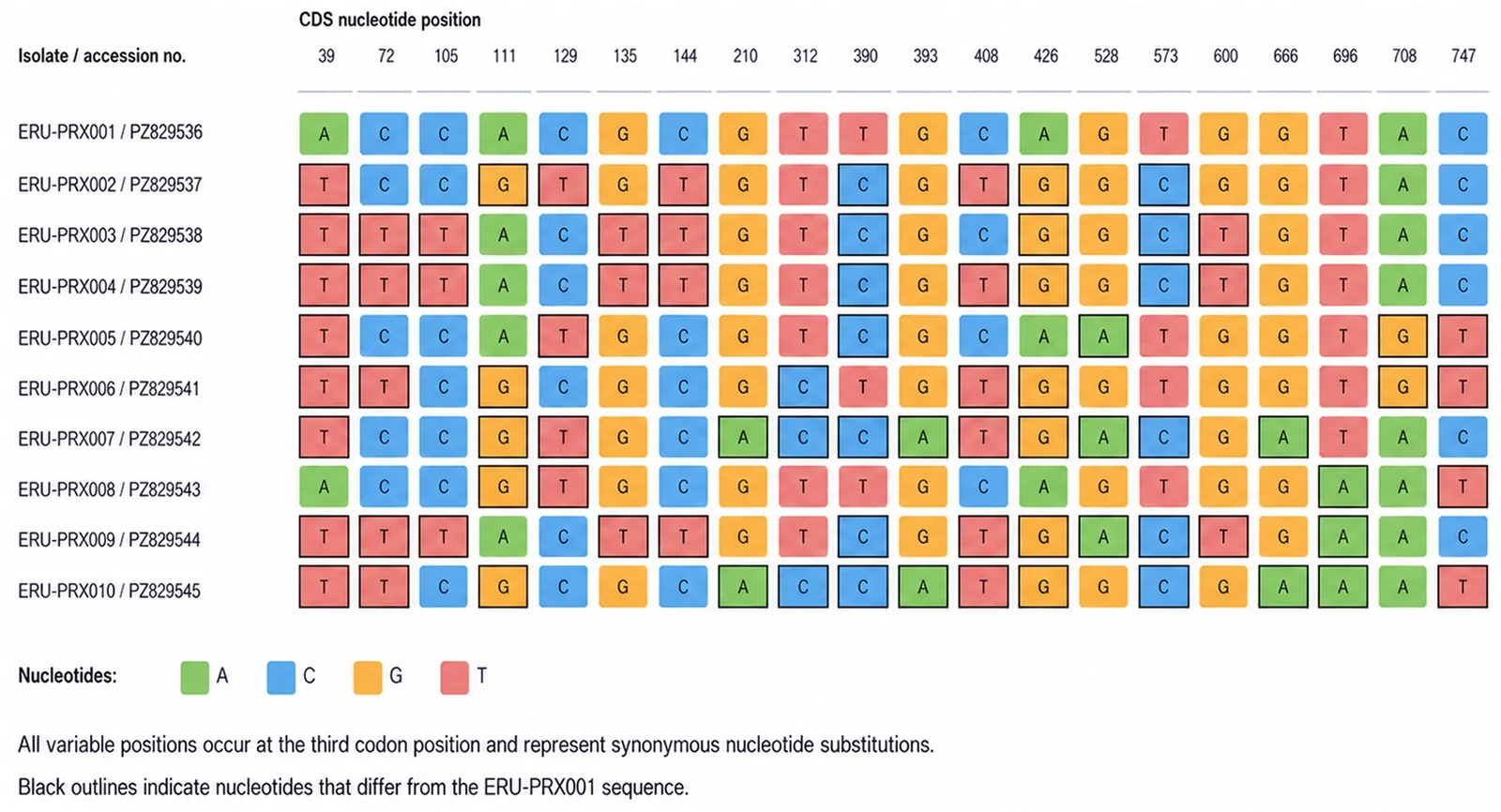
Variable nucleotide positions in *Prx2* coding sequences from 10 Turkish *Aedes albopictus* isolates. Colored cells identify the nucleotide present at each of the 20 polymorphic sites, and black outlines indicate nucleotides that differ from the ERU-PRX001 sequence. All variable sites occur at third codon positions and represent synonymous substitutions.

### Protein Features and Phylogenetic Placement

The predicted physicochemical properties of Prx2 were evaluated using the ERU-PRX001 protein sequence. This sequence encoded a 249-amino-acid protein with a predicted molecular mass of 28.115 kDa, a theoretical isoelectric point of 6.792, an instability index of 33.03, an aliphatic index of 86.91, and a grand average of hydropathicity (GRAVY) value of −0.196. For comparison, the orthologous aedine Prx2 proteins had closely similar predicted properties (*Aedes aegypti* XP_021696772.1: isoelectric point 7.15, instability index 33.6, aliphatic index 85.4, GRAVY −0.226; *Ochlerotatus camptorhynchus* XP_065089208.1: isoelectric point 6.50, instability index 35.0, aliphatic index 88.6, GRAVY −0.154), indicating that the ERU-PRX001 values are typical for mosquito 2-Cys Prxs and consistent with a stable (instability index < 40), moderately hydrophilic protein. Four cysteine residues were present at positions 12, 29, 100, and 221. The complete ERU-PRX001 protein showed 99.20% amino acid identity to the *Aedes albopictus* reference peroxiredoxin-2 protein XP_029725869.1.

Based on the NCBI Conserved Domain Database annotation of XP_029725869.1 and its projection onto the ERU-PRX001 sequence, the typical 2-Cys peroxiredoxin homologous region extended from residues 57 to 228. The projected peroxidatic and resolving cysteines occurred at positions 100 and 221, respectively. The homologous region contained the conserved FTFVCPTEI motif at residues 96–104, the GGLG motif at residues 143–146, and the EVCPAGW motif at residues 219–225. The characteristic C-terminal YF motif was located at residues 242–243, downstream of the annotated homologous region (Fig. 3).

**Fig. 3.**
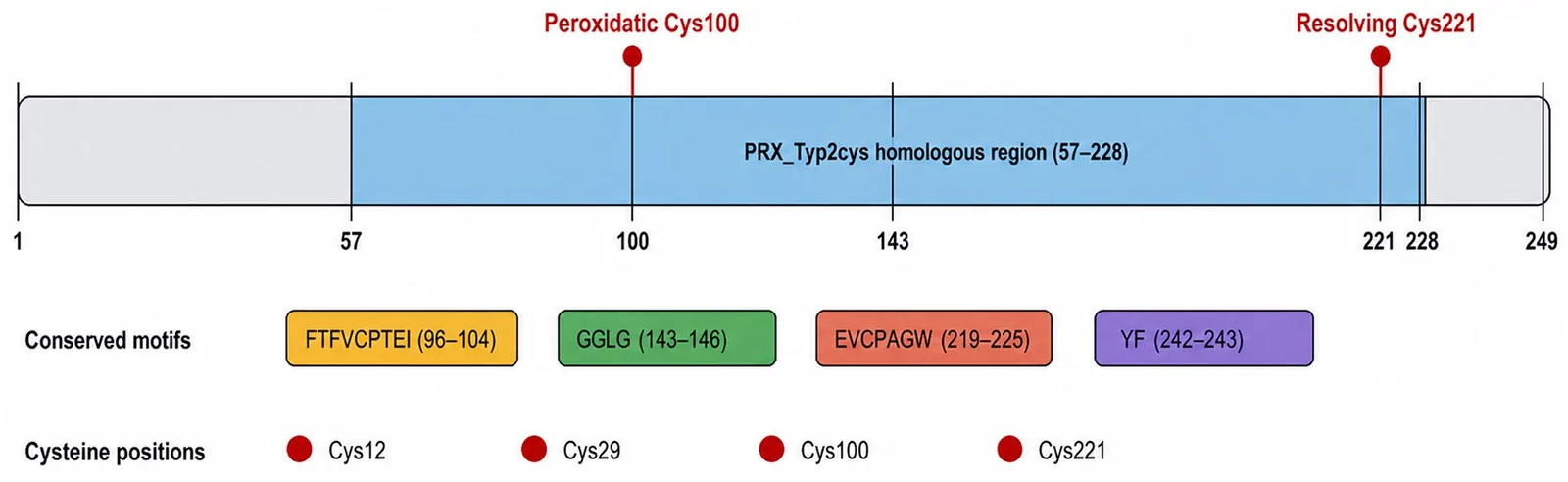
Structural and conserved-motif organization of the ERU-PRX001 (PZ829536) 2-Cys peroxiredoxin protein. The typical 2-Cys peroxiredoxin homologous region extends from residues 57 to 228 and contains the projected peroxidatic Cys100 and resolving Cys221, together with the conserved FTFVCPTEI, GGLG, and EVCPAGW motifs. The C-terminal YF motif is located at residues 242–243, downstream of the annotated homologous region. Domain boundaries and functional cysteine positions were projected from the NCBI Conserved Domain Database annotation of the closely related *Aedes albopictus* reference protein XP_029725869.1.

The 172-amino-acid homologous region was identical among all Turkish isolates and showed 100% identity to the corresponding region of the *Aedes albopictus* reference protein. No insertions or deletions were detected in the alignment. Amino acid identity across this region was 97.09% with *Aedes aegypti*, 96.51% with *Ochlerotatus camptorhynchus*, 95.93% with *Culex quinquefasciatus*, *Culex pipiens pallens*, and *Toxorhynchites rutilus septentrionalis*, and 93.02–93.60% with the three *Anopheles* species. Thirteen variable amino acid positions were identified across the 19 taxa. Nevertheless, Cys100, Cys221, and the characteristic FTFVCPTEI, GGLG, and EVCPAGW motifs were conserved in all sequences (Fig. 4).

**Fig. 4.**
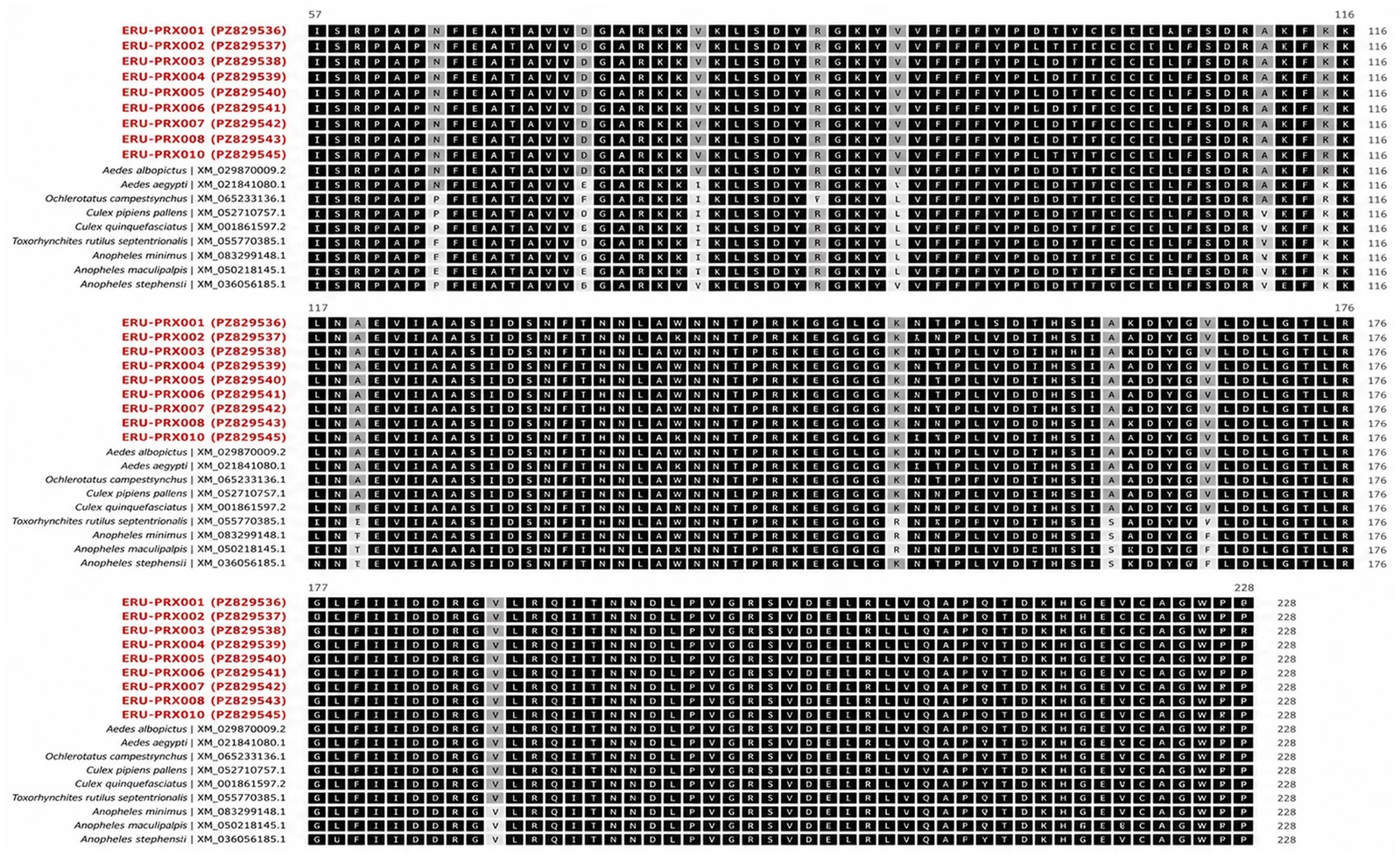
Multiple amino acid sequence alignment of the typical 2-Cys peroxiredoxin homologous region from Turkish *Aedes albopictus* isolates and reference mosquito species. The alignment spans residues 57–228 of ERU-PRX001 and comprises 172 amino acids. Dark shading indicates complete conservation, whereas gray or light shading indicates variable positions.

The midpoint-rooted bootstrap consensus tree inferred from the 516-nucleotide codon alignment grouped all 10 Turkish isolates with the *Aedes albopictus* reference sequence in a species-level cluster with 100% bootstrap support (Fig. 5). Support for relationships within this cluster was generally low, consistent with the 10 variable sites present among the isolates in this region. ERU-PRX001 and ERU-PRX008, which were identical across the 516-bp homologous region, grouped together with 85% bootstrap support. ERU-PRX007 and ERU-PRX010 also grouped together, with 72% bootstrap support, and ERU-PRX003, ERU-PRX004, and ERU-PRX009 grouped with 50% support. The *Aedes albopictus* cluster and *Aedes aegypti* formed a broader *Aedes* clade with 84% bootstrap support, to which *Ochlerotatus camptorhynchus* was joined with 83% support. *Culex quinquefasciatus* and *Culex pipiens pallens* formed a distinct clade with 100% support. The three *Anopheles* sequences also formed a monophyletic group with 100% support, within which *Anopheles stephensi* and *Anopheles maculipalpis* grouped with 61% support. No clearly resolved clustering corresponding to the geographic origins of the Turkish isolates was observed.

**Fig. 5.**
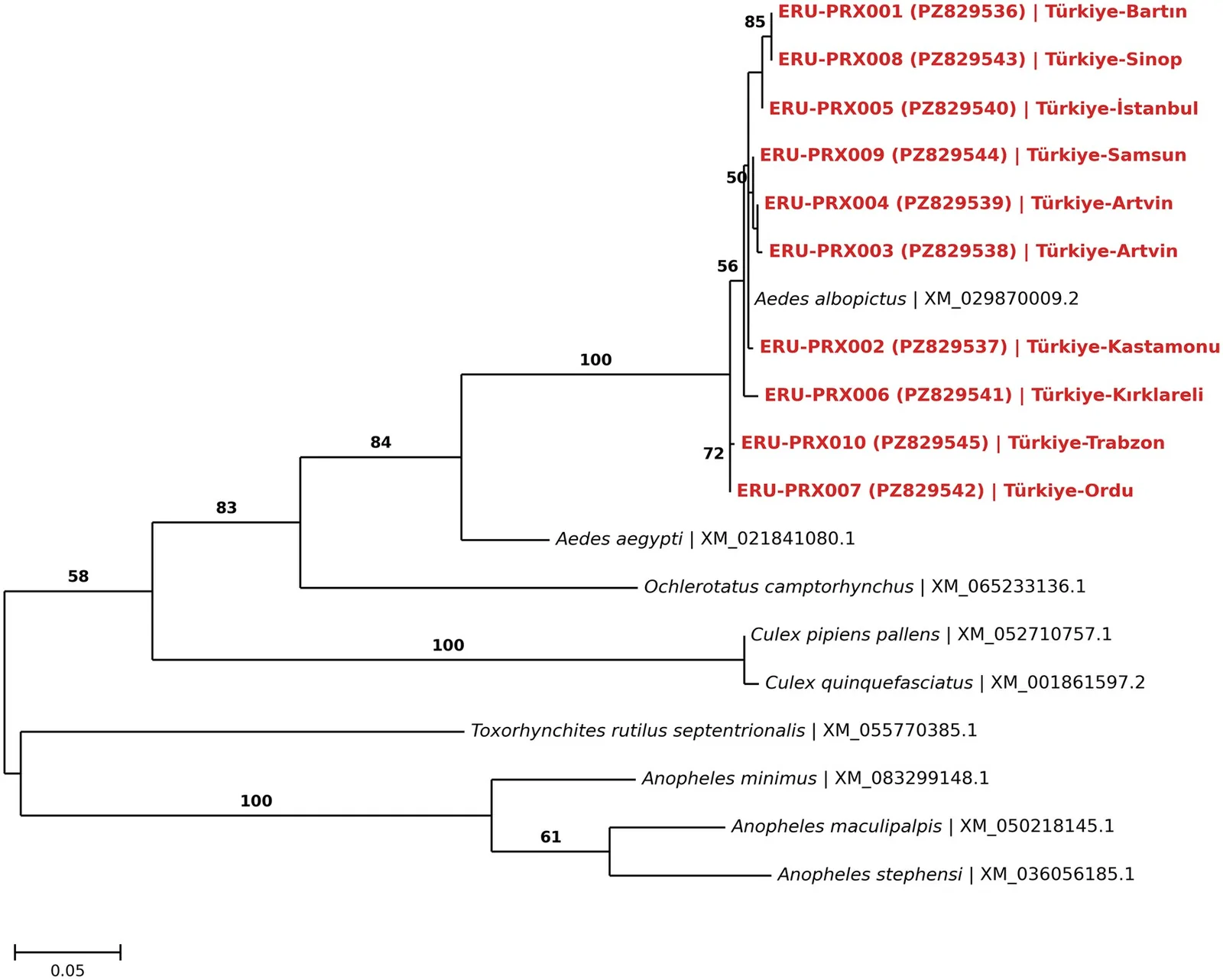
Midpoint-rooted maximum-likelihood bootstrap consensus tree of *Prx2* sequences from Turkish *Aedes albopictus* isolates and reference mosquitoes. The tree was inferred from a 516-nucleotide codon alignment using TNe + I for codon positions 1 + 2 and TPM2 + F + G4 for codon position 3 and is shown as the extended majority-rule consensus of 1,000 standard nonparametric bootstrap replicates, with branch lengths optimized by maximum likelihood on the original alignment. Bootstrap values ≥50% are shown on the corresponding branches and were mapped to bipartitions after midpoint rooting; the value on the root-adjacent branch refers to the bipartition divided by the root. Turkish isolates are shown in bold type. The scale bar represents 0.05 substitutions per nucleotide site.

### Recombinant Protein and Peroxidase Activity

The full-length *Prx2* coding sequence from ERU-PRX001 (PZ829536) was used for recombinant protein production. PCR screening with the T7 forward and gene-specific reverse primers generated the expected approximately 980-bp amplicon from the pET-28a(+)-Prx2 construct, whereas no amplification was detected in the no-template control. The identity and integrity of the construct were subsequently confirmed by Sanger sequencing.

The ERU-PRX001 coding sequence encoded a 249-amino-acid Prx2 polypeptide with a predicted molecular mass of 28.115 kDa, excluding the 6 × His tag and any vector-derived residues. Following induction with 0.1, 0.5, or 1.0 mM IPTG, SDS-PAGE revealed a prominent soluble protein band with an apparent molecular mass of approximately 28 kDa. The corresponding band was not prominent in the uninduced control. Nickel-affinity purification yielded a dominant product at the same apparent molecular mass. Immunoblot analysis of 25 ng of purified protein using an anti-6 × His antibody detected a distinct immunoreactive band, confirming production of the His-tagged recombinant Prx2 protein (Fig. 6).

**Fig. 6.**
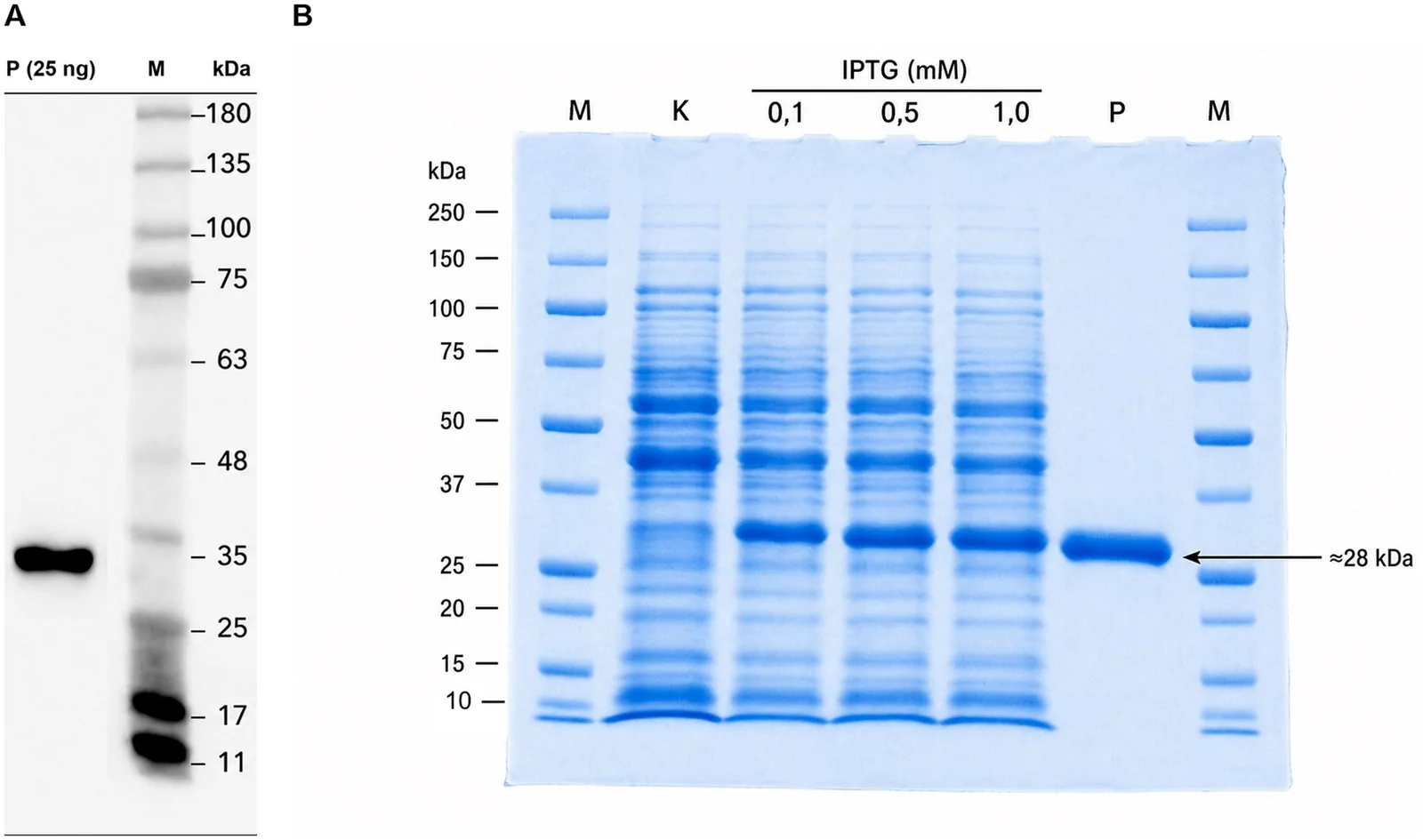
Western blot and SDS-PAGE analyses of recombinant Prx2 derived from ERU-PRX001 (PZ829536). (A) Anti-6 × His immunoblot of 25 ng of purified recombinant protein. (B) Coomassie Brilliant Blue-stained SDS-PAGE of soluble protein fractions from the uninduced control (K), cultures induced with 0.1, 0.5, or 1.0 mM IPTG, and the nickel-affinity-purified protein (P). M, molecular-mass marker. The arrow indicates the recombinant product with an apparent molecular mass of approximately 28 kDa.

The H_2_O_2_-removing activity of purified recombinant Prx2 increased with protein concentration and was markedly enhanced by DTT (Fig. 7). No H_2_O_2_ removal was detected in the protein-free controls (0 µg/ml), either in the presence or absence of DTT. In reactions containing 10 mM DTT, mean H_2_O_2_ removal was 2.50% ± 0.36%, 12.00% ± 0.70%, 24.00% ± 1.04%, 40.00% ± 1.37%, and 58.00% ± 2.77% at Prx2 concentrations of 5, 25, 50, 75, and 100 µg/ml, respectively. In the absence of DTT, the corresponding values were 0.50% ± 0.10%, 2.00% ± 0.26%, 4.00% ± 0.35%, 5.50% ± 0.30%, and 6.00% ± 0.36%. Values are presented as the mean ± SD of three independent experiments. At 100 µg/ml, H_2_O_2_ removal in the presence of DTT was approximately 9.7-fold higher than that measured without DTT.

**Fig. 7.**
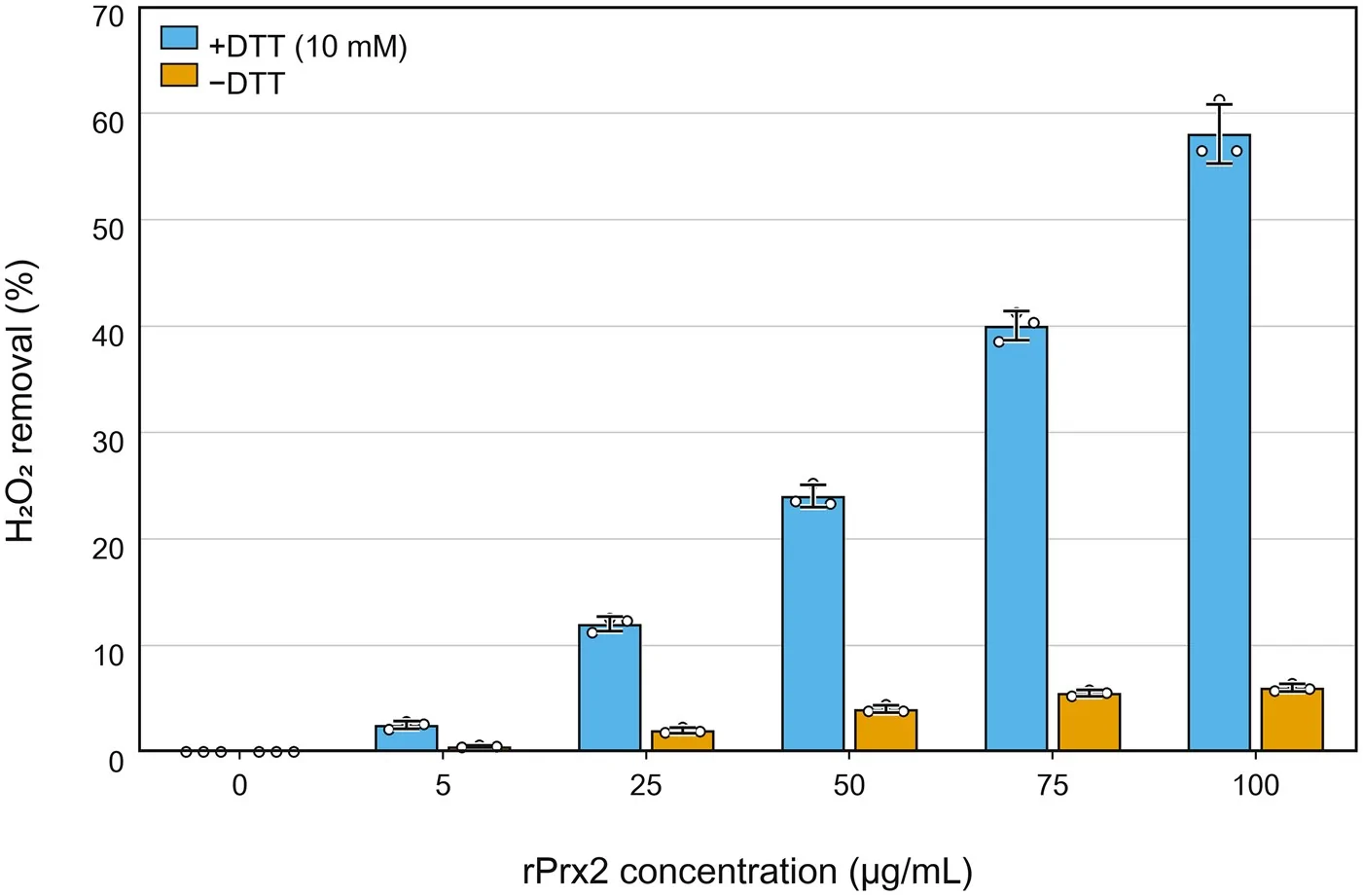
H_2_O_2_-removing activity of purified recombinant *Aedes albopictus* Prx2 in the presence or absence of 10 mM DTT. Protein concentrations of 0, 5, 25, 50, 75, and 100 µg/mL were evaluated. Bars represent means ± SD from three independent experiments, and open circles indicate the individual experiment means.

Two-way ANOVA, including the 0 µg/ml protein-free control as the sixth concentration level, detected significant effects of protein concentration (*F*_5__,__24_ = 977.500, *P *< 0.001), DTT condition (*F*_1__,__24_ = 3600.577, *P *< 0.001), and their interaction (*F*_5__,__24_ = 633.192, *P *< 0.001). The significant interaction demonstrated that the effect of increasing Prx2 concentration on H_2_O_2_ removal differed according to the presence or absence of DTT.

### Developmental and Pyriproxyfen-Responsive Expression

Basal *Prx2* expression differed significantly among developmental stages (*F*_6__,__1__4_ = 135.124, *P *< 0.001; Fig. 8). Relative expression remained similar across the four larval stages, with mean values ranging from 0.987 to 1.033. Expression decreased to its lowest level in pupae (0.417 ± 0.035) and subsequently increased in adult males (1.583 ± 0.123) and adult females (1.783 ± 0.165). Tukey-adjusted comparisons placed L1–L4 in the same statistical group, pupae in a separate group, and both adult sexes in a third group. Thus, pupal expression was significantly lower than larval expression, whereas expression in both adult sexes was significantly higher than that in the larval and pupal stages. Although the mean value was numerically higher in adult females than in adult males, the difference between the sexes was not significant.

**Fig. 8.**
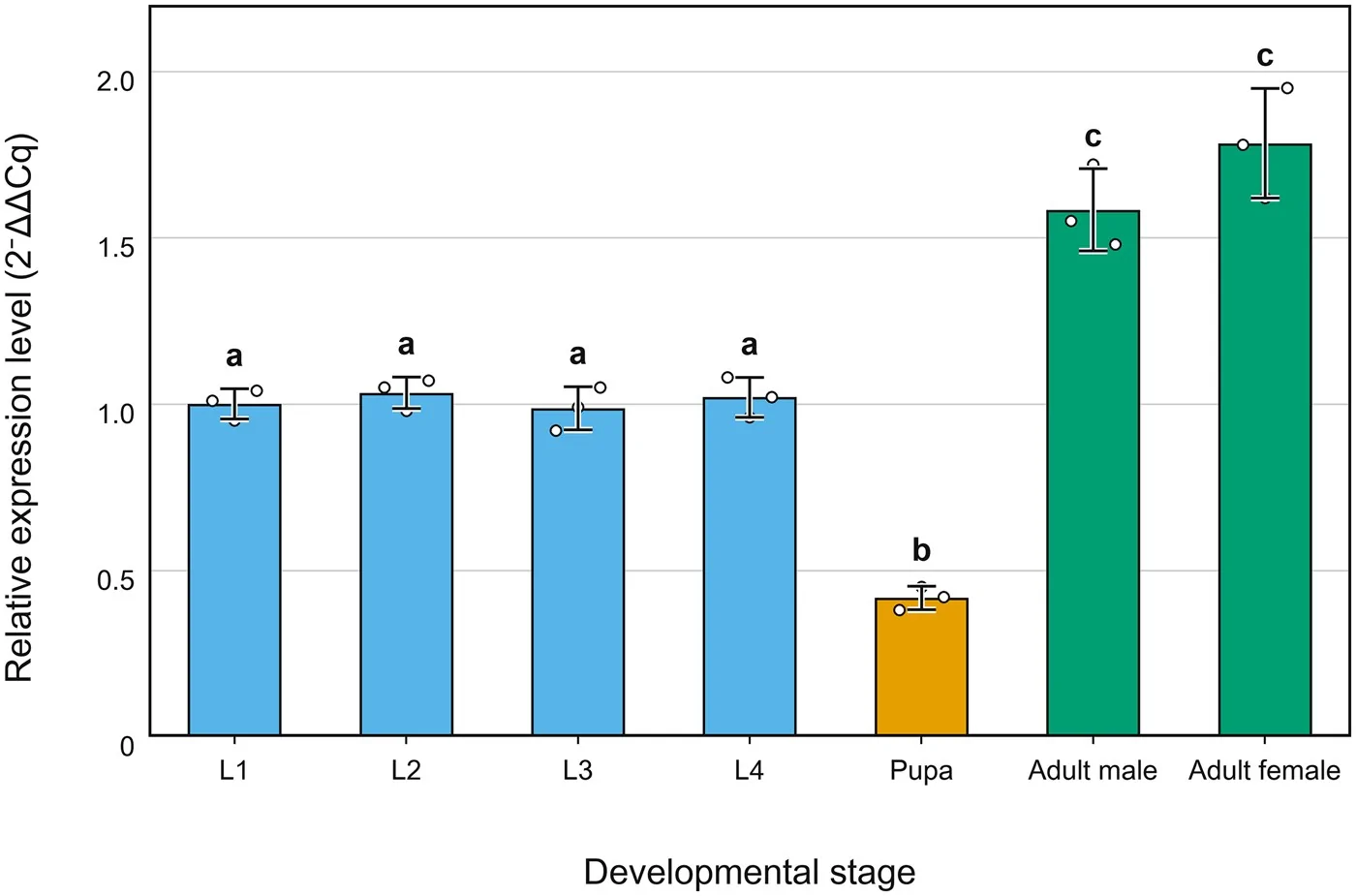
Relative *Prx2* expression across developmental stages of *Aedes albopictus*. Bars represent mean 2^−^ΔΔCq values ± SD from three independent biological replicates, and open circles indicate individual biological replicates. Different letters indicate significant differences among developmental stages based on Tukey-adjusted comparisons. Statistical analyses were performed using the corresponding ΔCq values.

The effect of pyriproxyfen on *Prx2* expression varied among developmental stages, as indicated by significant effects of stage (*F*_4__,__20_ = 196.245, *P *< 0.001), treatment (*F*_1__,__20_ = 219.428, *P *< 0.001), and the stage × treatment interaction (*F*_4__,__20_ = 51.875, *P *< 0.001; Fig. 9). Because the interaction was significant, the effect of pyriproxyfen was evaluated separately at each developmental stage using prespecified contrasts with Holm adjustment.

**Fig. 9.**
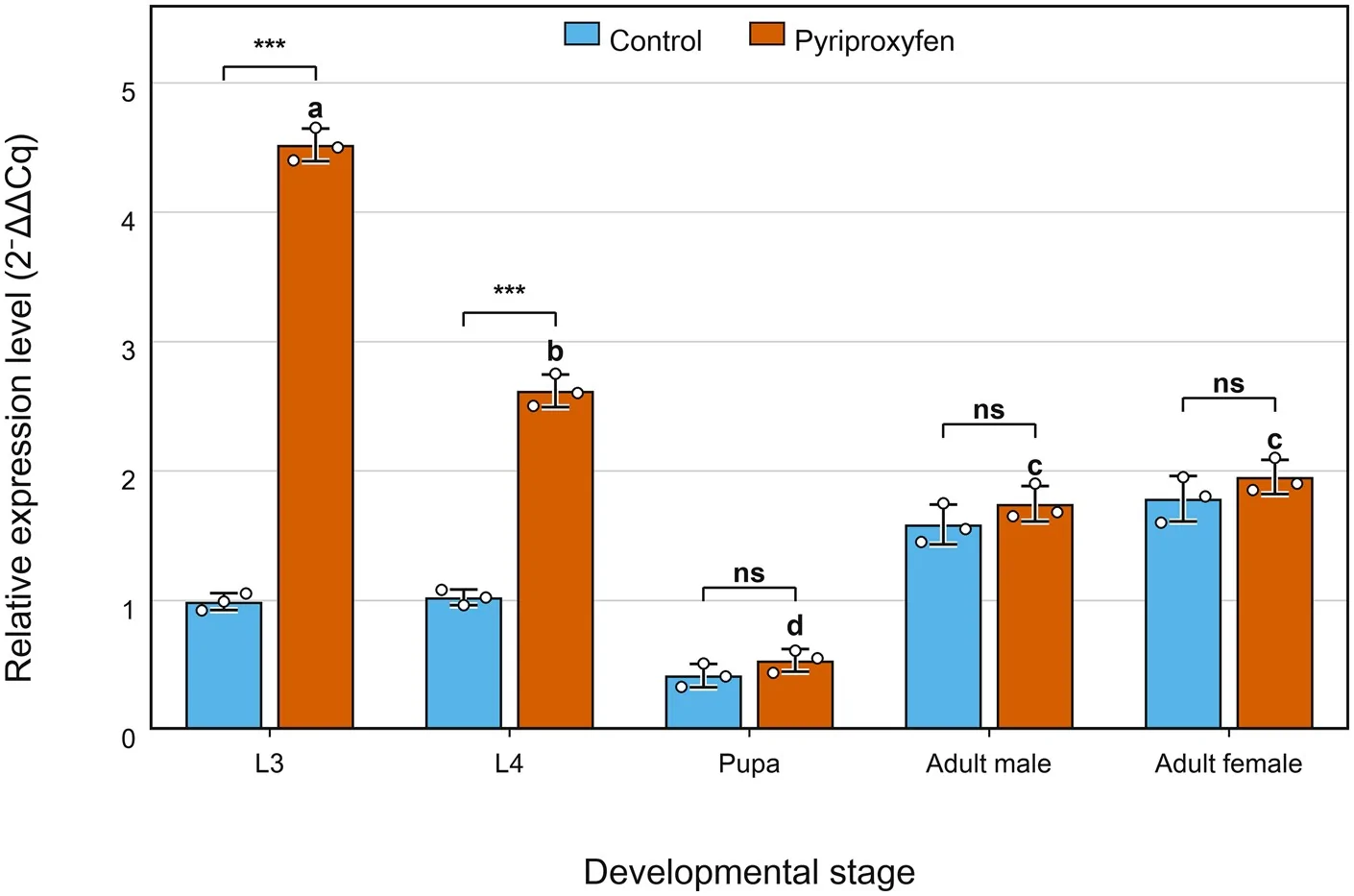
Relative *Prx2* expression across developmental stages following exposure to 0.06 ppb pyriproxyfen. Bars represent mean 2^−^ΔΔCq values ± SD from three independent biological replicates, and open circles indicate individual biological replicates. Asterisks indicate significant differences between pyriproxyfen-exposed and stage-matched control groups based on prespecified contrasts with Holm adjustment (***P < 0.001); ns indicates no significant difference. Different letters above the pyriproxyfen-exposed groups indicate Tukey-adjusted differences among exposed developmental stages. Later-stage samples comprised individuals that survived the initial L3 exposure. Statistical analyses were performed using the corresponding ΔCq values.

At L3, mean relative expression increased from 0.987 ± 0.065 in the control group to 4.517 ± 0.126 following exposure to 0.06 ppb pyriproxyfen, corresponding to an approximately 4.58-fold increase (Holm-adjusted *P* < 0.001). At L4, expression increased from 1.020 ± 0.060 in controls to 2.617 ± 0.126 in the pyriproxyfen group, representing an approximately 2.57-fold increase (Holm-adjusted *P* < 0.001). In contrast, no statistically detectable treatment-related differences were observed among the surviving pupae (control: 0.417 ± 0.090; pyriproxyfen: 0.533 ± 0.086), adult males (control: 1.583 ± 0.153; pyriproxyfen: 1.743 ± 0.137), or adult females (control: 1.783 ± 0.176; pyriproxyfen: 1.950 ± 0.132; Holm-adjusted *P* = 0.554 for each comparison). Thus, treatment-associated increases were detected in L3 and L4 larvae at the sampled time points, whereas the later-stage survivor groups did not differ statistically from their stage-matched controls.

Relative-expression values are presented as 2^−^ΔΔCq means ± SD from three independent biological replicates, whereas all statistical tests were performed using the corresponding ΔCq values.

## Discussion

This study integrates intraspecific sequence variation, protein characterization, recombinant enzyme activity, and stage-specific transcription to characterize Prx2 in *Aedes albopictus*. The complete coding sequences contained 20 polymorphic sites and represented 10 distinct haplotypes, but all substitutions occurred at third codon positions and were synonymous. Consequently, all Turkish isolates encoded an identical 249-amino-acid protein. This combination of nucleotide diversity and amino acid conservation is consistent with functional constraint on the Prx2 protein, although formal inference of purifying selection would require a larger multilocus dataset and explicit analysis of synonymous and nonsynonymous substitution rates. The occurrence of distinct haplotypes among the sampled isolates is compatible with the mitogenomic diversity and multiple maternal lineages recently reported for *Ae. albopictus* in the Black Sea region of Türkiye (Diop et al. 2026). Nevertheless, the present data comprise a single coding locus and generally one isolate per locality; they therefore cannot independently resolve introduction history or fine-scale population structure.

The phylogenetic analysis placed the Turkish Prx2 sequences with the *Ae. albopictus* reference and separated them from the other mosquito taxa examined. This placement, which received 100% bootstrap support, supports the taxonomic identity of the recovered sequences. However, the low support for most relationships within this cluster and the limited length of the 516-nucleotide alignment constrain more detailed phylogeographic interpretation. The supported subgroups observed among some Turkish isolates reflect sequence similarity at this locus but should not be regarded as geographically structured populations. Additional isolates per locality and rapidly evolving nuclear or genome-wide markers would be required to evaluate population connectivity and regional differentiation.

Conservation of peroxidatic Cys100, resolving Cys221, and the FTFVCPTEI, GGLG, EVCPAGW, and C-terminal YF motifs supports classification of ERU-PRX001 as a typical 2-Cys peroxiredoxin. In this enzyme family, the peroxidatic and resolving cysteines mediate peroxide reduction and intermolecular disulfide formation, whereas the GGLG and YF motifs contribute to the conformational transitions associated with catalysis and are characteristic of many peroxide-sensitive eukaryotic Prxs (Hall et al. 2009, Perkins et al. 2015). However, the presence of these motifs alone does not demonstrate susceptibility to hyperoxidation; direct assessment of Prx oxidation states would be required to establish that property in *Ae. albopictus* Prx2.

Purified recombinant Prx2 removed H_2_O_2_ in a concentration-dependent manner, and this activity was markedly enhanced by DTT. The strong dependence on a reducing thiol is consistent with the catalytic cycle of a typical 2-Cys Prx, in which the oxidized enzyme must be reduced before repeated catalytic turnover. A closely comparable pattern was reported for recombinant thioredoxin peroxidase 1 from *Apis cerana cerana*, which displayed concentration-dependent H_2_O_2_ removal in the presence of DTT but little activity without the reductant (Yu et al. 2011). The present assay therefore supports thiol-dependent peroxide-removing activity of recombinant *Ae. albopictus* Prx2. DTT is, however, an artificial reducing agent, and the endpoint ferrithiocyanate assay does not provide catalytic constants or identify the physiological electron donor. Future experiments should reconstitute the *Ae. albopictus* thioredoxin–thioredoxin reductase–NADPH system, determine substrate specificity and kinetic parameters, and examine the redox and oligomeric states of Prx2 during catalysis.

Basal Prx2 transcript abundance varied markedly across development. Expression remained similar from L1 through L4, decreased to its lowest level in pupae, and increased in adult males and females. Developmental and tissue-dependent Prx expression has been reported across diverse insects and other arthropods, although the direction and magnitude of these changes differ among species and Prx isoforms (Yu et al. 2011, Kusakisako et al. 2016, Yang et al. 2025). For example, *A. cerana cerana* thioredoxin peroxidase expression varies among larval, pupal, and adult stages, while tick Prx2 expression changes among tissues and in response to blood feeding (Yu et al. 2011, Kusakisako et al. 2016). The lower pupal expression observed here may be associated with the substantial physiological reorganization accompanying metamorphosis, whereas higher adult expression could reflect increased requirements related to flight, reproduction, or environmental exposure. These explanations remain hypotheses because whole-body RNA was analyzed. Tissue-specific measurements in the midgut, fat body, ovaries, and flight muscle, together with protein-level analysis, will be required to identify the biological processes responsible for the developmental pattern.

The treatment-associated difference in *Prx2* transcription varied among the sampled developmental stages. *Prx2* expression increased approximately 4.6-fold at L3 and 2.6-fold at L4, whereas no statistically detectable treatment-related difference was observed among the surviving pupae or adults. Because later-stage samples necessarily consisted only of survivors, this pattern permits at least two explanations that cannot be separated by the present design: the transcriptional response may have attenuated during development, or survival after exposure may have altered the composition of the later-stage groups, for example, if individuals with stronger or more persistent responses were less likely to complete development. The absence of detectable differences in pupae and adults therefore does not establish stage-specific silencing or the absence of a transstadial effect. Independent exposures initiated at each developmental stage, or experiments linking individual survival with transcriptional responses, would be required to distinguish these alternatives. A directionally similar response has been reported in adult *Apis cerana cerana*, in which pyriproxyfen treatment induced an approximately 2.3-fold increase in thioredoxin peroxidase 1 transcription, with peak expression occurring 2 h after treatment (Yu et al. 2011). That comparison demonstrates pyriproxyfen-responsive transcription of an insect thioredoxin peroxidase, but the magnitude and timing cannot be compared directly because the studies differed in species, developmental stage, dose, exposure route, and sampling schedule.

Pyriproxyfen acts through juvenile-hormone signaling and can alter the expression of endocrine and metabolic genes. In *Aedes aegypti*, pyriproxyfen modifies Methoprene-tolerant-dependent signaling, 20-hydroxyecdysone-responsive transcription, and metabolic gene expression (Ahmed et al. 2020). In adult *Ae. albopictus*, nonlethal exposure disrupts reproductive performance and expression of genes involved in vitellogenesis and hormonal regulation (Kancharlapalli and Brelsfoard 2024). These observations provide a plausible endocrine and metabolic context for the early Prx2 response detected here. However, the present experiment did not measure ROS concentrations, oxidative damage, Prx2 protein abundance, or Prx2 redox state. The transcriptional increase is therefore consistent with, but does not establish, a compensatory antioxidant response to pyriproxyfen-induced oxidative or metabolic stress. Similarly, the absence of significant differences in pupae and adults indicates that a persistent transcriptional effect was not detected at the sampled time points; it does not exclude shorter-lived responses between sampling points or persistent changes in other redox-related genes.

The Prx2 response should not be interpreted as evidence of pyriproxyfen resistance. Sublethal pyriproxyfen exposure has been associated with altered susceptibility to other insecticides in *Anopheles arabiensis*, and field populations of *Ae. albopictus* with reduced susceptibility to pyriproxyfen have been reported (Opiyo et al. 2021, Lin et al. 2024). Nevertheless, establishing a contribution of Prx2 to resistance would require comparisons between susceptible and resistant populations, concentration–response experiments, functional manipulation of Prx2 expression, and simultaneous measurement of multiple antioxidant and detoxification pathways. The present results demonstrate an exposure-responsive transcriptional phenotype but not a causal role in pyriproxyfen detoxification or resistance.

Several limitations define the scope of the conclusions. Sequence variation was evaluated in only 10 isolates and at a single coding locus, limiting population-level inference. Recombinant activity was measured with an artificial reductant in an endpoint assay rather than with the physiological thioredoxin system. Expression analyses were based on whole-body RNA from three biological replicates, a single pyriproxyfen concentration, and one sampling point within each developmental stage. qPCR interpretation is further limited by the absence of experimental amplification-efficiency estimates, the lack of quantitative RNA-integrity metrics, and use of *β-actin* as a single reference gene without stability validation across developmental stages or treatments. Consequently, the reported 2^−^ΔΔCq fold-change magnitudes should be interpreted cautiously. Moreover, later-stage samples necessarily represented individuals that survived the initial exposure. Consequently, the lack of a statistically detectable treatment difference in pupae and adults cannot distinguish genuine developmental attenuation from selective survival of individuals with lower or shorter-lived transcriptional responses. Transcript abundance was not accompanied by measurements of *Prx2* protein, enzyme activity in vivo, ROS accumulation, or oxidative damage. These limitations do not invalidate the observed transcriptional patterns but restrict their quantitative, mechanistic, and transstadial interpretation.

In conclusion, *Ae. albopictus Prx2* showed substantial synonymous nucleotide variation while retaining complete amino acid conservation among the Turkish isolates examined. Its conserved catalytic features and thiol-dependent H_2_O_2_-removing activity support its identity as a functional typical 2-Cys peroxiredoxin. *Prx2* transcript abundance increased in L3 and L4 larvae following exposure to a nominal pyriproxyfen concentration of 0.06 ppb, whereas no statistically detectable treatment-related differences were observed among the surviving pupae or adults at the sampled time points. Because this concentration was not independently validated as sublethal in the present colony and later-stage sampling was restricted to survivors, the results support a pyriproxyfen-associated larval transcriptional response under the tested conditions but do not establish a strictly stage-limited or nonpersistent transstadial response. The findings provide a basis for determining whether redox regulation contributes functionally to the physiological effects of juvenile hormone analogs in invasive mosquitoes.

## Supplementary Material

ieag119_Supplementary_Data

## Data Availability

The *Prx2* coding sequences generated in this study have been deposited in GenBank under accession numbers PZ829536–PZ829545.
