# Supplementary material for: Molecular and functional characterization of a typical 2-Cys peroxiredoxin and its developmental and pyriproxyfen-responsive expression in Aedes albopictus (Diptera: Culicidae)
Source: J Insect Sci. 2026 Oct 27;26(5):ieag119. doi: 10.1093/jisesa/ieag119 (PMC13713186; doi:10.1093/jisesa/ieag119)
Supplement: Supplementary file 1 [file ieag119_supplementary_data.docx]

**Supplementary Tables**

Sequence recovery and intraspecific variation of the *Prx2* coding sequence in *Aedes albopictus*

**Supplementary Table S1. Isolate metadata and GenBank accession numbers for 10 *Aedes albopictus* *Prx2* coding sequences.**

| **Sequence code** | **Source sample code** | **Geographic origin** | **Sex** | **CDS length (bp)** | **Predicted protein length (aa)** | **GenBank accession no.** |
| --- | --- | --- | --- | --- | --- | --- |
| ERU-PRX001 | ABC14AeM | Bartın | Male | 750 | 249 | PZ829536 |
| ERU-PRX002 | ABC2AeF | Kastamonu | Female | 750 | 249 | PZ829537 |
| ERU-PRX003 | ART3AeM | Artvin | Male | 750 | 249 | PZ829538 |
| ERU-PRX004 | ART6AeF | Artvin | Female | 750 | 249 | PZ829539 |
| ERU-PRX005 | IST21AeF | İstanbul | Female | 750 | 249 | PZ829540 |
| ERU-PRX006 | KRK18AeF | Kırklareli | Female | 750 | 249 | PZ829541 |
| ERU-PRX007 | OG9AeF | Ordu | Female | 750 | 249 | PZ829542 |
| ERU-PRX008 | SS19AeF | Sinop | Female | 750 | 249 | PZ829543 |
| ERU-PRX009 | SS4AeF | Samsun | Female | 750 | 249 | PZ829544 |
| ERU-PRX010 | TR21AeM | Trabzon | Male | 750 | 249 | PZ829545 |

**Note.** All GenBank records comprise 750-bp coding sequences encoding predicted 249-amino-acid proteins.

**Supplementary Table S2**

**General FASTQ quality control and Prx2-specific read-support metrics**

**A.** Overall FASTQ sequencing and filtering statistics.

| **Sequence code** | **Raw reads (n)** | **Raw bases (Gb)** | **Mean read length R1/R2 (bp)** | **Raw Q20/Q30 (%)** | **Raw GC (%)** | **Filtered reads (n)** | **Filtered bases (Gb)** | **Filtered Q20/Q30 (%)** | **Filtered GC (%)** | **Reads retained (%)** |
| --- | --- | --- | --- | --- | --- | --- | --- | --- | --- | --- |
| ERU-PRX001 | 69,926,268 | 9.467 | 135/135 | 95.59/89.75 | 40.96 | 66,792,630 | 9.186 | 96.19/90.50 | 40.79 | 95.52 |
| ERU-PRX002 | 74,442,288 | 9.763 | 131/131 | 95.89/90.40 | 40.93 | 70,496,264 | 9.442 | 96.49/91.13 | 40.74 | 94.70 |
| ERU-PRX003 | 193,042,334 | 25.961 | 134/134 | 95.94/90.42 | 41.11 | 184,216,546 | 25.234 | 96.47/91.07 | 40.96 | 95.43 |
| ERU-PRX004 | 206,905,804 | 28.121 | 135/135 | 95.67/89.97 | 41.10 | 197,661,452 | 27.290 | 96.28/90.74 | 40.96 | 95.53 |
| ERU-PRX005 | 95,629,430 | 12.967 | 135/135 | 95.86/90.25 | 41.08 | 91,669,632 | 12.629 | 96.37/90.88 | 40.98 | 95.86 |
| ERU-PRX006 | 85,371,522 | 11.347 | 132/132 | 96.16/90.73 | 41.07 | 81,689,798 | 11.063 | 96.61/91.28 | 40.95 | 95.69 |
| ERU-PRX007 | 66,464,594 | 8.737 | 131/131 | 95.84/89.58 | 41.21 | 63,365,242 | 8.518 | 96.22/90.03 | 41.05 | 95.34 |
| ERU-PRX008 | 189,349,502 | 25.532 | 134/134 | 95.26/88.66 | 41.75 | 181,113,180 | 24.835 | 95.78/89.29 | 41.58 | 95.65 |
| ERU-PRX009 | 59,012,904 | 8.092 | 137/137 | 95.79/90.15 | 41.09 | 56,667,476 | 7.870 | 96.37/90.87 | 40.94 | 96.03 |
| ERU-PRX010 | 346,172,630 | 45.687 | 131/131 | 96.09/90.74 | 41.51 | 329,099,898 | 44.372 | 96.62/91.39 | 41.36 | 95.07 |

**Note.** Values were obtained from the fastp reports generated for each paired-end library. Read counts refer to individual reads, not read pairs. Q20 and Q30 are the percentages of bases with Phred quality scores of at least 20 and 30, respectively. Reads retained (%) was calculated as filtered reads divided by raw reads × 100.

**Supplementary Table S2 continued**

**B.** Prx2-specific target/decoy mapping and strict CDS coverage.

| **Sequence code** | **Target primary reads** | **Decoy primary reads** | **Target duplicates (%)** | **Unique target MAPQ ≥30** | **Unique decoy MAPQ ≥30** | **Safe fragments / rescued reads** | **CDS depth mean/median** | **CDS depth min-max** | **CDS bases ≥5×/≥10×** | **Strict callable bases (n/750)** |
| --- | --- | --- | --- | --- | --- | --- | --- | --- | --- | --- |
| ERU-PRX001 | 11,381 | 41,432 | 47.7 | 4,575 | 5,133 | 2,993/5,545 | 46.59/48.0 | 12–100 | 750/750 | 750/750 |
| ERU-PRX002 | 13,788 | 44,189 | 49.5 | 5,439 | 5,563 | 3,396/6,512 | 53.54/51.5 | 15–140 | 750/750 | 750/750 |
| ERU-PRX003 | 30,549 | 117,475 | 62.2 | 7,581 | 12,353 | 5,209/9,557 | 71.32/74.8 | 10–135 | 750/750 | 750/750 |
| ERU-PRX004 | 32,531 | 131,493 | 61.3 | 8,679 | 13,463 | 5,352/10,118 | 77.64/81.0 | 10–161 | 750/750 | 750/750 |
| ERU-PRX005 | 15,053 | 58,416 | 53.0 | 5,713 | 7,342 | 3,818/7,030 | 54.84/51.3 | 14–125 | 750/750 | 750/750 |
| ERU-PRX006 | 15,001 | 49,429 | 53.2 | 5,909 | 6,289 | 3,928/7,375 | 56.58/55.9 | 10–114 | 750/750 | 750/750 |
| ERU-PRX007 | 12,769 | 39,319 | 49.3 | 4,665 | 4,808 | 2,863/5,212 | 39.73/36.9 | 13–76 | 750/750 | 750/750 |
| ERU-PRX008 | 32,567 | 109,143 | 66.0 | 9,473 | 10,485 | 5,920/11,538 | 66.68/62.5 | 12–178 | 750/750 | 750/750 |
| ERU-PRX009 | 9,086 | 35,778 | 45.8 | 4,018 | 4,403 | 2,618/4,939 | 41.22/40.5 | 12–100 | 750/750 | 750/750 |
| ERU-PRX010 | 64,029 | 198,639 | 70.5 | 13,102 | 18,663 | 8,762/16,483 | 110.60/102.6 | 10–269 | 750/750 | 750/750 |

**Note.** Reads were mapped with minimap2 to the Prx2 target transcript (XM_029870009.2), with the *Aedes albopictus* reference genome (GCF_006496715.1) included to divert paralogous and off-target (decoy) reads. Target and decoy primary-read counts were determined before duplicate and mapping-quality filtering. Target duplicate percentage is the marked-duplicate fraction among target primary reads. Unique target and decoy counts exclude marked duplicates and require MAPQ ≥30. Safe fragments are read pairs anchored by a unique target read after decoy filtering; rescued reads are the individual reads contained in these fragments, including mates recovered through pair anchoring. CDS depth statistics were calculated over the 750 CDS positions from the reads that passed the consensus-calling filters (duplicate-excluded, MAPQ ≥30, base quality ≥30). A position was considered strictly callable when it had ≥10× depth, a major allele frequency ≥0.80, and support for the called allele from at least three reads on each strand; positions that failed these criteria were not filled from the reference. All CDS coordinates refer to the 750-bp Prx2 coding sequence.
